# Anti-Hu Paraneoplastic Polyradiculoneuropathy Masquerading As Guillain-Barré Syndrome: A Case Report

**DOI:** 10.7759/cureus.96296

**Published:** 2025-11-07

**Authors:** Sarah I Zahid, Bushra Alnuaimi, Nadia I Zahid, Ans A Mahmood, Tasnim Moustafa

**Affiliations:** 1 Internal Medicine, College of Medicine, Gulf Medical University, Ajman, ARE; 2 Internal Medicine, Gulf Medical University, Ajman, ARE; 3 Family Medicine, Nassau University Medical Center, East Meadow, USA

**Keywords:** anna-1, anti-hu, guillain-barré syndrome mimic, paraneoplastic syndrome, small cell lung carcinoma

## Abstract

Paraneoplastic neurological syndromes (PNS) can precede the detection of an underlying malignancy and often mimic more common neurological conditions, leading to significant diagnostic delays. This report describes a case of anti-Hu (ANNA-1)-associated PNS that closely mimicked Guillain-Barré syndrome (GBS). A 47-year-old male presented with acute ascending flaccid weakness following a respiratory infection. Cerebrospinal fluid analysis demonstrated albuminocytologic dissociation, supporting an initial diagnosis of GBS. The patient showed a poor clinical response to intravenous immunoglobulins. Further investigation identified a right upper lobe lung mass, which biopsy confirmed as small-cell lung carcinoma. The presence of high-titer anti-Hu antibodies (1:7680) established a definitive diagnosis of paraneoplastic polyradiculoneuropathy. Despite aggressive treatment with chemotherapy, immunotherapy, and plasma exchange, his neurological status deteriorated, culminating in hypercapnic respiratory failure necessitating tracheostomy. His hospital course was further complicated by iatrogenic osmotic demyelination syndrome secondary to rapid sodium correction. The patient ultimately died from his condition. This case underscores that a treatment-resistant, GBS-like presentation may be the initial manifestation of a severe paraneoplastic syndrome. A lack of response to standard immunomodulatory therapy should prompt an expedited search for an occult malignancy and associated onconeural antibodies, as the PNS itself often dictates a grave prognosis. Additionally, this case highlights the heightened vulnerability of such patients to serious iatrogenic complications from routine medical management.

## Introduction

Paraneoplastic neurological syndromes (PNS) represent a group of immune-mediated disorders that may manifest prior to or concurrently with the diagnosis of an underlying cancer, most frequently small-cell lung carcinoma (SCLC). Syndromes associated with anti-Hu antibodies (ANNA-1) are characterized by a spectrum of neurological presentations, which can include subacute or progressive sensory, sensorimotor, or motor neuropathies. These are often accompanied by significant pain and autonomic dysfunction. Although the most recognized presentation is a subacute sensory neuronopathy, acute-onset variants that closely resemble Guillain-Barré syndrome (GBS) have been documented, posing a significant challenge to timely and accurate diagnosis [1-6].

The underlying mechanism of neuronal injury in anti-Hu syndromes is primarily mediated by T-cell-driven inflammation, leading to axonal and neuronal degeneration. This pathophysiology results in a characteristically poor response to immunomodulatory treatments that target humoral immunity, such as IV immunoglobulin and plasma exchange. This stands in contrast to the typically favorable therapeutic response observed in classic GBS. The detection of anti-Hu antibodies is considered highly specific for a paraneoplastic origin, and their identification necessitates investigation for an occult malignancy, particularly SCLC, even in the absence of a prior cancer diagnosis [2,4-6].

The clinical outcome for patients with anti-Hu-associated neurological syndromes is generally unfavorable. The degree of neurological disability frequently becomes the principal determinant of survival, often outweighing the impact of the tumor burden itself. Common complications include chronic electrolyte imbalances, such as hyponatremia, and unremitting neurological deterioration. This case serves to illustrate the diagnostic and management challenges in anti-Hu paraneoplastic polyradiculoneuropathy, especially when the initial clinical picture is indistinguishable from GBS and further complicated by metabolic disturbances and progressive neurological decline [1-6].

## Case presentation

A 47-year-old male was admitted in May 2025, presenting with a several-day history of progressive, ascending weakness and numbness in all four limbs. This neurological onset occurred shortly after a recent hospitalization for community-acquired pneumonia. The patient's functional capacity deteriorated significantly, leading to an inability to walk and grasp objects. Comprehensive neurological assessment revealed flaccid quadriparesis with generalized areflexia. CSF analysis demonstrated albuminocytologic dissociation, characterized by elevated protein concentration (2.06 g/L) with a normal white cell count (10 leukocytes/μL). Electrophysiological studies were consistent with a demyelinating polyneuropathy. Pulmonary function testing (Table 1) identified significant respiratory compromise, manifesting as a severe restrictive ventilatory pattern with markedly reduced diffusion capacity (diffusing capacity of the lung for carbon monoxide (DLCO) 35% of predicted). These collective findings supported a preliminary diagnosis of GBS, prompting administration of a five-day IV immunoglobulin protocol. The therapeutic response proved unsatisfactory, with minimal clinical improvement observed.

**Table 1:** Pulmonary function test results at initial presentation. FVC: Forced Vital Capacity; FEV₁: Forced Expiratory Volume in One Second; FEV₁/FVC: Ratio of Forced Expiratory Volume in One Second to Forced Vital Capacity; DLCO: Diffusing Capacity of the Lung for Carbon Monoxide; KCO: Transfer Coefficient for Carbon Monoxide (DLCO corrected for alveolar volume); VA: Alveolar Volume.

Parameter	Predicted	Measured	% Predicted	Interpretation
Spirometry				
FVC (L)	4.11	3.02	73%	Mild restriction
FEV₁ (L)	3.37	2.4	71%	Mild restriction
FEV₁/FVC (%)	78.75	79.44	101%	Normal
Diffusion capacity				
DLCO (mL/min/mmHg)	9.53	3.33	35%	Severe reduction
KCO (DLCO/VA)	1.5	0.77	52%	Moderate reduction
VA (L)	6.19	4.3	69%	Mild reduction

**Figure 1:**
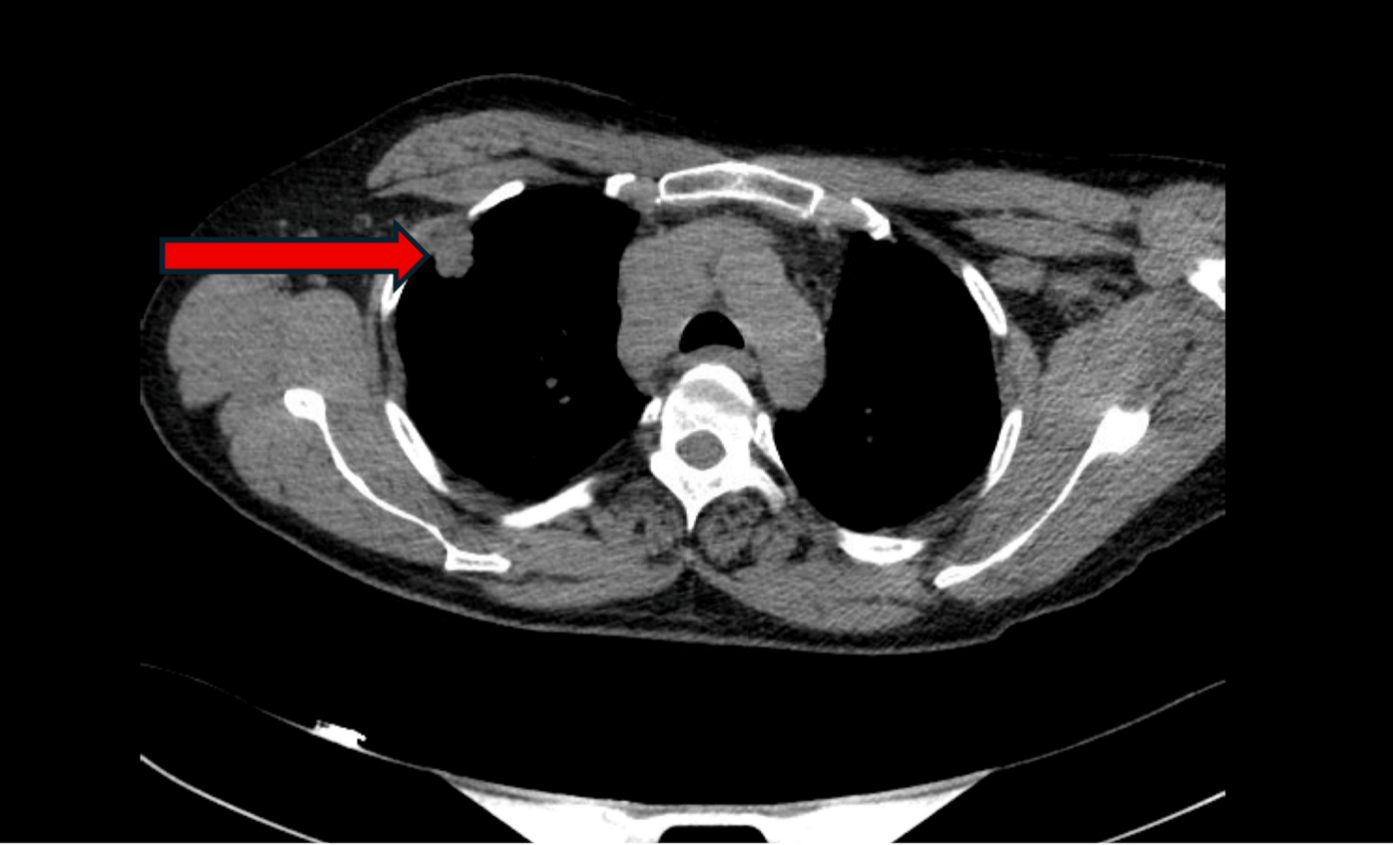
CT thorax with contrast showing a pleural-based soft-tissue opacity in the right upper lobe measuring 2.0 cm (red arrow), which was biopsied and confirmed to be small-cell lung carcinoma.

The inadequate response to conventional immunotherapy prompted further diagnostic investigation. A CT scan of the thorax demonstrated a right upper lobe pulmonary nodule with concomitant mediastinal lymphadenopathy (Figure 1). Histopathological analysis of CT-guided biopsy specimens confirmed small-cell lung carcinoma. Subsequent serological profiling detected high-titer anti-Hu (ANNA-1) antibodies (1:7680), establishing the definitive diagnosis of anti-Hu paraneoplastic sensorimotor polyradiculoneuropathy.

Multimodal oncological therapy was initiated, including carboplatin, etoposide, and atezolizumab. Despite this comprehensive regimen and additional immunomodulatory interventions, including repeated IV immunoglobulin and five cycles of therapeutic plasma exchange, his neurological status continued to decline progressively. The clinical course was further complicated by acute hypercapnic respiratory failure, requiring advanced ventilatory support and subsequent tracheostomy. During intensive care management, correction of chronic paraneoplastic syndrome of inappropriate antidiuretic hormone secretion (SIADH)-induced hyponatremia with hypertonic saline precipitated iatrogenic osmotic demyelination syndrome, with characteristic MRI findings (interval development of central pontine fluid-attenuated inversion recovery (FLAIR) hyperintensity). The patient's clinical status continued to deteriorate, leading to his passing from progressive neurological and respiratory failure.

## Discussion

This case illustrates a classic yet challenging clinical scenario in which a paraneoplastic syndrome perfectly mimicked a more common neurological disorder, leading to a delay in identifying the underlying pathology. The discussion centers on three critical learning points.

First, this case highlights the concept of the “Guillain-Barré mimic,” where a paraneoplastic process can simulate GBS. The initial presentation of this patient was classic for GBS [7]. However, the patient’s lack of improvement following a standard five-day IV immunoglobulin protocol constituted a major diagnostic pivot point. Such treatment refractoriness should trigger an immediate and thorough investigation for alternative etiologies, specifically through comprehensive onconeural antibody serology and rigorous malignancy screening [8]. The subsequent identification of anti-Hu antibodies, which demonstrate high specificity for paraneoplastic origins and a strong association with SCLC, effectively excluded idiopathic GBS as the diagnostic entity [9].

Second, establishing the diagnosis of an anti-Hu-associated paraneoplastic syndrome carries profound prognostic implications. In contrast to the typically monophasic and often recoverable course of GBS, anti-Hu neuropathies are characterized by severe, axonal-predominant injury that is relentlessly progressive [10]. The neuronal damage is frequently irreversible at the time of diagnosis, accounting for the characteristically poor response to immunomodulatory interventions, as evidenced by this patient’s failure to improve despite both IVIG and plasma exchange. The neurological disability associated with this paraneoplastic syndrome often dictates mortality more significantly than the tumor burden itself, with respiratory failure from neuromuscular weakness being a principal determinant of survival [11]. This prognostic reality is crucial for setting realistic goals of care with patients and families.

Third, this case underscores the importance of supportive care and the management of iatrogenic risk. The emergence of osmotic demyelination syndrome represented a devastating yet avoidable complication. The management of chronic hyponatremia secondary to SIADH mandates a carefully controlled correction protocol, generally limiting serum sodium elevation to no more than 6-8 mmol/L per 24-hour period, to prevent dangerous osmotic fluid shifts that can damage pontine and extrapontine structures [12]. The administration of hypertonic saline in this context signifies a critical failure in supportive care and serves as a key learning point in managing complex, chronically ill patients.

## Conclusions

This tragic outcome underscores critical lessons for clinical practice. Firstly, any acute neuropathy resembling GBS that fails to respond to initial immunotherapy must be considered a potential paraneoplastic disorder. This mandates an immediate and thorough search for an underlying cancer, as the neurological syndrome often precedes the oncological diagnosis. Secondly, a confirmed anti-Hu paraneoplastic neuropathy carries a devastating prognosis, in which the neurological damage, not the tumor itself, often becomes the primary driver of mortality. This reality necessitates honest and timely conversations with patients and families to align care with realistic outcomes. Finally, the management of these complex patients requires extreme vigilance, particularly in avoiding iatrogenic catastrophes such as osmotic demyelination. Ultimately, improving outcomes hinges on three principles: maintaining a high index of suspicion for malignancy in treatment-resistant cases, pivoting diagnostics swiftly when standard therapies fail, and providing meticulous supportive care to prevent secondary complications.
